# Pathogens That Rewrite the Rules: Ascoviruses, Elegant Manipulators of Cell Death Pathways and Architects of the Extracellular Viral Paradigm

**DOI:** 10.3390/pathogens14111094

**Published:** 2025-10-27

**Authors:** Sarah R. Rudd, Leticia S. Miranda, Sharon J. Asariah, Chloe S. Rodgers, Jenive T. Estrada, Michael A. Alonzo, Dennis K. Bideshi

**Affiliations:** 1 Department of Biological Sciences, California Baptist University, Riverside, CA 92504, USA; srudd@calbaptist.edu (S.R.R.); leticia_miranda@berkeley.edu (L.S.M.); sharon.asariah@md.cusm.edu (S.J.A.); jenivethi.estrada@calbaptist.edu (J.T.E.); malonzo@calbaptist.edu (M.A.A.); 2 Integrated Biomedical Graduate Studies, School of Medicine, Loma Linda University, Loma Linda, CA 92350, USA; 3 Division of Infectious Diseases and Vaccinology, School of Public Health, University of California, Berkeley, CA 94720, USA; 4 School of Medicine, California University of Science and Medicine, Colton, CA 92324, USA; 5 Department of Chemical Sciences, California Baptist University, Riverside, CA 92504, USA; chloes.rodgers@calbaptist.edu

**Keywords:** lepidoptera, *Ascoviridae*, *Ascovirus*, *Toursvirus*, Nucleocytoplasmic Large double-stranded DNA Viruses (NCLDV), viral caspase, apoptosis, virion-containing vesicles, endoparasitoid wasp, ceramide-sphingosine-1-phosphate rheostat, extracellular viral paradigm

## Abstract

Ascoviruses (AVs) are obligate intracellular pathogens that target the larval and pupal stages of lepidopteran insects, specifically moth caterpillars. AVs are unique among viruses in their (i) transmission mode, (ii) gross pathology, (iii) virion ultrastructure, (iv) genomic architecture featuring a remarkable combination of genes, and (v) ability to reprogram host cell death and lipid biosynthetic pathways to generate virion-containing vesicles (VCVs). The metabolically active acellular VCVs are repurposed to complete virogenesis and to facilitate dissemination by endoparasitoid wasps. Since their discovery in the late 20th century, research has focused on these distinctive traits and, to a lesser extent, their potential for biological control. Among AV proteins are the large DNA-binding P64 family, inhibitor of apoptosis-like proteins (IAPs), executioner caspase and caspase-like proteins, and lipid-modifying enzymes, which together drive their novel cytopathology. This review synthesizes current knowledge of AV biology and proposes a framework for understanding VCV formation by integrating predicted viral protein functions with host cellular pathways, including the ceramide–sphingosine-1-phosphate rheostat and apoptosis.

## 1. Introduction

AVs are large circular dsDNA viruses transmitted among lepidopteran moth larvae (caterpillars) by hymenopteran endoparasitoid wasps [1,2]. Recognized by the ICTV since the establishment of the *Ascoviridae* family in 2002 [3,4], AVs remain understudied when compared to other insect viruses, particularly baculoviruses that are well described from basic and applied perspectives [5,6,7,8]. Among all viruses, AVs stand out due to their distinctive ultrastructure, transmission mechanism, gross pathology, and cytopathology, features that highlight a unique association among caterpillars, AVs, and parasitoid wasps [9,10,11].

**Figure 1:**
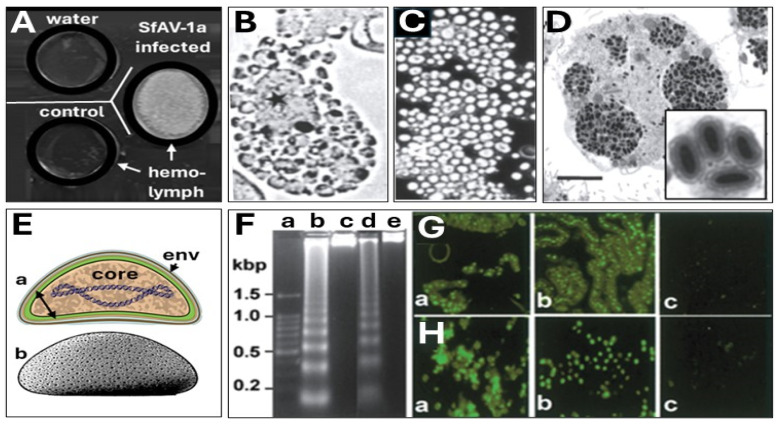
Pathology induced by SfAV-1a in vivo and in vitro. (**A**) Milky white hemolymph collected 7 days post-infection from fourth instar *Spodoptera exigua* (*Se*) larvae. (**B**) Infected fat body cell cleaving into ~20–50 viral vesicles at 72 h post-infection; vesicle formation proceeds inward from the plasma membrane. The central area (star) has not yet formed vesicles. (**C**) Hemolymph containing > 10^8^ viral vesicles/mL at 120 h post-infection. (**D**) Electron micrograph of a viral vesicle showing clustered virions; inset shows virions at higher magnification. (**E**) (**a**) Simplified structure of an AV virion with envelope, inner particle, and dsDNA genome; reproduced with permission from ViralZone (SIB Swiss Institute of Bioinformatics (https://viralzone.expasy.org/preview_by_species/276, accessed on 27 September 2025, under CC BY 4.0.) (**b**) Artistic rendering of a negatively stained AV virion showing a reticulated surface typical of AVs. (**F**) DNA fragmentation in SfAV-1a-infected *S. frugiperda* cell (SF21) and *Se* fat body tissue, compared to controls; (**a**) standard DNA markers; DNA isolated from (**b**) SfAV-1a-infected SF21 cells at 12 h post-infection, (**c**) uninfected SF21 cells; (**d**) SfAV-1a-infected and (**e**) uninfected *Se* larvae. (**G**) TUNEL assay showing extensive apoptosis in *Se* (**a**) infected fat body tissue, (**b**) uninfected tissue treated with DNase I to induce chromosomal fragmentation, and (**c**) untreated control. (**H**) TUNEL assay of SF21 cells (**a**) 12 h after infection with SfAV-1a, (**b**) DNase I-treated and (**c**) mock-infected control. Bars, 1 μm. Panels (**B**–**D**,**F**–**H**) adapted from Bideshi et al. *Genes Dev.* **2005**, *19*, 1416–1421 [12]; panel (**E**) (**b**) based on Federici, *Proc. Natl. Acad. Sci. USA* **1983**, *80*, 7664–7668 [15].

The cytopathic effects induced by AVs in caterpillars and cultured cells are particularly striking (Figure 1). Upon infection, AVs trigger marked cellular hypertrophy, followed by an orchestrated disassembly of the nuclear and plasma membranes. This process culminates in the formation of specialized acellular bioactive VCVs within which virogenesis continues [1,2]. It relies on the manipulation of host-regulated cell death (RCD) pathways, particularly apoptosis and pyroptosis [12,13]. Pyroptosis is thought to serve as a strategy to suppress apoptosis and enhance viral replication during the early phase of infection (3–24 h), before the apoptotic program becomes activated and modified in later stages (48–120 h). In contrast to most viruses that inhibit RCD to preserve host cell viability and support replication [14], AVs actively induce and modulate death pathways to facilitate virogenesis. Their molecular toolkit includes inhibitor of apoptosis proteins (IAPs), cathepsins, lipid-modifying enzymes, caspase-like proteins, and in SfAV-1a, an executioner caspase [12], all of which contribute to the production of VCVs.

VCVs are a vital component of the tripartite system (endoparasitoid wasp-AV-caterpillar) and are essential for the ecological persistence of AVs. During egg laying (oviposition), endoparasitic wasps contaminate their ovipositors with VCVs through direct contact with the caterpillar hemolymph, enabling the transfer of AVs to new hosts during subsequent oviposition events. Because AVs do not replicate within the wasp, the insect serves as a mechanical vector. This transmission route is unique to endoparasitoids; ectoparasitoids, which lay eggs externally, do not vector AVs [1,2]. Although baculoviruses and iridoviruses can also be incidentally transmitted by parasitoids, they lack the specialized vesicle-based delivery mechanism that is characteristic of AVs [1,2].

Over the past 45 years, foundational studies have revealed key aspects of AV biology, including transmission, pathology, and virion ultrastructure [1,2,15]. Yet, the most compelling frontier remains the elucidation of the molecular mechanisms underlying their atypical cytopathology. With at least 11 AV genomes now sequenced and annotated, and recent host–virus transcriptomic analyses shedding light on gene expression dynamics [16,17,18,19], new insights into the complex tripartite system are emerging. These advances are beginning to clarify how AVs manipulate host cellular pathways to orchestrate virogenesis and immune evasion. This review synthesizes current knowledge of AV biology and offers theoretical perspectives on their unique features, aiming to spark broader interest not only among insect pathologists and virologists, but also among researchers exploring novel host–pathogen interactions and ecology, and the evolution of symbiotic viral systems.

## 2. Discovery, Classification and Evolutionary Origin

AVs are likely widespread in nature due to their association with ubiquitous braconid and ichneumonid wasp vectors, yet they remained undetected until the late 1970s. Their initial identification in noctuid caterpillars (*Scotogramma trifolii*, *Trichoplusia ni*, and *Helicoverpa zea*) occurred in Southern California [1]. The chronic and subtle progression of AV infections, even in terminal stages, made field detection challenging. However, a distinctive symptom, i.e., milky-white hemolymph (Figure 1A), renders AV infection uniquely recognizable [1,2,12]. These early observations, along with the later identification of Diadromus pulchellus toursvirus (DpTV-1a) (formally DpAV-4a), led to the formal establishment of the family *Ascoviridae* [3,4,15,20,21].

According to the International Committee on the Taxonomy of Viruses (ICTV), AVs are classified within the Realm *Varidnaviria*, Kingdom *Bamfordvirae*, Phylum *Nucleocytoviricota*, Class *Megaviricetes*, Order *Pimascovirales*, and Family *Ascoviridae*, which includes two genera, *Ascovirus* and *Toursvirus* [4]. The genus *Ascovirus* comprises three species (SfAV-1a, TnAV-2a, and HvAV-3a), while the genus *Toursvirus* currently includes DpTV-1a. Expansion of the species lists and host range is anticipated, as metagenomic analyses have yielded full and partial genome sequences of DjAV-2a from the *Dasineura jujubifolia* fly [22] and Diabotrica TV-3a from *Diabrotica undecimpuntata howardi* and *Diabrotica barberi* corn root beetles [23].

To date, at least nine AV and two TV genomes have been sequenced (Table 1). TVs possess smaller genomes (119.3–142.6 kbp) compared to AVs (157–200 kbp), with G+C content ranging from ~45.4–49.3%, and 123–194 predicted coding sequences. Among AVs, at least 40 genes are conserved [4]. Early phylogenetic analyses of DNA polymerase sequences revealed a close relationship between AVs and invertebrate iridoviruses (IIVs), despite differences in virion morphology, genome structure, and host pathology. Iridoviruses are icosahedral and classified into *Alphairidovirinae* (vertebrate-infecting) and *Betairidovirinae* (invertebrate-infecting), whereas AVs are bacilliform or allantoid and infect moth caterpillars exclusively [24].

Further comparative studies placed AVs within the Nucleocytoplasmic Large DNA Viruses (NCLDVs) [25,26], a group that includes seven recognized families (*Phycodnaviridae*, *Mimiviridae*, *Ascoviridae*, *Iridoviridae*, *Marseilleviridae*, *Asfarviridae*, and *Poxviridae*), alongside several unclassified or proposed lineages such as Faustovirus, Kaumoebavirus, Klosneuvirus, Medusavirus, Mollivirus, Pithovirus, and Pandoravirus. These viruses have genomes ranging from ~106 kbp to over 2474 kbp, encoding between 99 and 2541 open reading frames (ORFs), and share a conserved core of approximately 50 genes [27,28].

Consensus phylogenetic trees based on sequences of major capsid protein, DNA polymerase, thymidine kinase, and ATPase III from algal viruses (*Phycodnaviridae)*, iridoviruses (*Iridoviridae*), and African swine fever virus (ASFV, *Asfarviridae*) indicate that AVs are most closely related to Chilo iridescent virus (CIV, IIV6). Comparative genomic analysis further revealed that SfAV-1a, the type species of AVs [29], shares approximately 20% of its protein orthologs with IIV6, while exhibiting lower similarity to vertebrate iridoviruses, phycodnaviruses, and ASFV [1,22,23,25,30,31]. Finally, phylogenetic studies of 46 protein orthologs suggest that TVs may be more closely aligned with insect iridoviruses than AVs [23].

### Unique Features of TVs Are Reminiscent of Polydnavirus Biology

Although TVs, such as DpTV-1a [21,32], are not the primary focus of this review, their biology offers compelling contrasts to AVs and intriguing parallels to polydnaviruses (PDVs; proposed family *Polydnaviriformidae*) [33]. PDVs are integral to a complex symbiosis involving hymenopteran endoparasitoid ichneumonid and brachonid wasps, lepidopteran larvae, and the virus [33,34,35,36]. Two PDV groups are recognized: brachoviruses (*Brachonidae*) and ichnoviruses (*Ichneumonidae*). Unlike AVs and TVs, PDVs have segmented genomes embedded within the wasp genome and replicate only in the calyx region of female wasp ovaries. During oviposition, PDVs are co-injected with the wasp egg into the host larva, where they do not replicate, but instead express virulence genes that suppress host immunity and manipulate development to favor parasitoid survival.

In contrast to the mechanical transmission of AVs, TVs exhibit a more intimate, PDV-like symbiosis. DpTV-1a replicates modestly in female wasp ovaries and is vertically transmitted via the egg [21,32]. Infected eggs are deposited into lepidopteran larvae within which the emerging parasitoid feeds. Upon maturation, the parasitoid either exits or pupates inside the host. This self-propagating autoinfection cycle ensures the persistence of TVs within wasp populations, highlighting their unique evolutionary strategy.

Unlike its lepidopteran host *Acrolepiopsis assectella*, DpTV-1a does not cause disease in the parasitoid wasp, *Diadromus pulchellus* (Hymenoptera: *Ichneumonidae*) [4,21]. Instead, it exists in a symbiotic relationship, persisting as an episome in various tissues (head, thorax, abdomen) and producing virions in the female reproductive tract [21,32]. DpTV-1a facilitates wasp larval development by suppressing the immune response of the lepidopteran host, creating a permissive environment for parasitoid survival. Viral genes encode immunosuppressive proteins that prevent the host hemocytes from attacking wasp eggs and larvae.

This immune suppression is evidenced by experiments showing nylon monofilament implants trigger encapsulation and melanization, hallmarks of insect innate immunity, in *A. assectella* [37,38,39]. However, these responses are significantly reduced in insects infected with DpTV-1a, indicating active suppression of host defenses. This is in contrast to the productive infection by SfAV-1a, TnAV-2, and HvAV-3, where the melanization response in the hemolymph persists for up to 10 days post-infection. The observed suppression of the prophenoloxidase (proPO) cascade, a central pathway in invertebrate immunity, is consistent with findings in other host-endoparasitoid systems [40,41,42,43,44]. The cascade involves the activation of PO (phenoloxidase), an enzyme critical for melanization, which plays a vital role in protecting insects against pathogens and tissue damage.

The biological similarities between TVs and PDVs suggest a shared evolutionary origin. Early phylogenetic analyses of the pox-D5 NTPase protein family suggested that TVs might represent an ancestral or intermediate form leading to ichnoviruses of the *Polydnaviridae* family [34,45]. However, more recent studies indicate that PDV genomes are derived from a range of endogenous viral elements, including nudiviruses [46,47]. Future identification and molecular characterization of novel TVs will be crucial to uncovering complex host–pathogen dynamics and to clarify their evolutionary history.

## 3. General Biology of AVs

The gross pathology caused by AVs has been well documented [48,49,50,51,52,53]. Early symptoms in noctuid larvae are subtle, but within 24 h, infected individuals show reduced feeding, stunted growth, and weight loss. Unlike healthy larvae, which can quadruple in size within 3–4 days, infected larvae exhibit arrested development, difficulty molting, bloated thorax, and a distinctive white or creamy discoloration of the body and hemolymph (Figure 1A), a hallmark of acute AV infection that is eventually lethal.

Remarkably, infected larvae often fail to progress beyond the third instar yet survive far longer than healthy counterparts. Survival duration varies by species: *Helicoverpa armigera* can persist up to 36 days post-infection, while *Spodoptera exigua* and *S. litura* survive up to 19 and 20 days, respectively [15,22,23,54,55,56]. This extended survival likely reflects evolved viral strategies to optimize replication, VCV biogenesis, and transmission efficiency via parasitoid wasps.

The ease of diagnosing AV disease by observing the milky hemolymph symptom without the aid of a microscope led to the discovery of new isolates across the U.S., France, Australia, Indonesia, China, Mexico, and Japan [34,52,53,57,58,59,60]. Laboratory studies show that *Crocidolomia pavonana* and *Plutella xylostella* can serve as semi-permissive hosts for HvAV-3e [61,62], suggesting AVs may infect species beyond noctuids, though their natural host range remains uncertain. Tissue tropism also varies: TnAV-2a and HvAV-3a infect the fat body, tracheal matrix, and epidermis, while SfAV-1a is largely restricted to the fat body [1,2].

## 4. AV Virion Ultrastructure

During virogenesis, mature virions begin to appear around 12 h post-infection, following nuclear rupture, and continue through the cleavage of the cell into VCVs (Figure 1B–D) [1,2,15]. The earliest recognizable structure is the multilaminar layer of the inner particle, composed of a unit membrane and an external layer of protein subunits. Simultaneously, a dense nucleoprotein core aggregates on the interior surface, completing inner particle formation. This core is then enveloped by membranes, likely synthesized de novo. Similar virion assembly and maturation processes occur in other large dsDNA viruses, such as herpesviruses, iridoviruses, and poxviruses, where structural components associate before differentiation [63,64].

Transmission electron micrographs show that AV virions are enveloped and structurally complex, with species-specific shapes, i.e., bacilliform in SfAV-1a and allantoid in TnAV-2a and HvAV-3a [1]. Despite morphological differences, their structural components are consistent: a DNA/protein core enclosed by an internal lipid bilayer and surrounded by a protein subunit layer. AV virions possess two lipid membranes, one with the inner particle and another forming the external envelope (Figure 1E(a)). In negatively stained preparations, they exhibit a distinctive reticulate pattern (Figure 1E(b)), likely due to the superimposed protein subunits on both membranes, setting them apart from other viral families [15].

Although the cytopathology and virogenesis of different AVs are essentially identical, both subtle and obvious differences are noted. Specifically, for TnAV-2 and HvAV-3, virions aggregate and form inclusion bodies toward the periphery of the vesicle, whereas for SfAV-1a, occlusion bodies form a “foamy” vesicular matrix consisting of a mixture of protein and minute spherical vesicles [1,54,65]. These occlusion bodies (OBs) are distinct from those produced by other types of viruses, including entomopoxvirus and the polyhedral or granular OBs of baculoviruses, in which enveloped virions are embedded in rigid matrices composed of viral-encoded polyhedrin and granulin proteins, respectively [63,66].

## 5. Proteomic Analysis of AV Virions

AV virion protein profiles vary across species, with TnAV-2c, SfAV-1a, and HvAV-3i encoding 7, 21, and 67 proteins, respectively [45,67,68]. Although 81 proteins were detected in HvAV-3i virion preparations, potential contamination by non-virion proteins cannot be excluded. Compared to TnAV-2c and SfAV-1a, other insect viruses possess more virion-associated proteins, for example, IIV6 has 44 [69]. Baculoviruses, considered to share evolutionary links to AVs [70], contain 23 to 73 virion proteins [71] and encode 100–200 total proteins, many with structural or functional roles in host cell invasion and replication. Entomopoxviruses typically have over 100 virion proteins involved in structure, replication, and host interactions [72,73,74].

### 5.1. Proteins and Peptides Shared by SfAV-1a, HvAV-31 and TnAV-2c Virions

Of 17 virion proteins or peptides shared by these three AVs [45,67,68], 6 were classified as hypothetical proteins as they do not contain discernible structural features to suggest functional roles, whereas 10 share structural domains or motifs with proteins available in the online databases [45,67,68] (Table 2; Supplementary Materials). We note that transcripts coding for these proteins have been detected in somatic and hemolymph tissues infected with SfAV-1a and TnAV-6a [17].

Viral-coded polymerases are essential for DNA replication and transcription, and in many instances these enzymes are components of infectious virions indicating their crucial role in viral biology immediately upon cellular invasion [109,110,111,112,113,114]. It is interesting that DNA-dependent DNA polymerases and DNA-dependent RNA polymerases are not present in AV virions, indicating that the genes coding for these enzymes are expressed early upon viral entry into the cell. Indeed, their corresponding genes are expressed 6 h to 7 days in somatic and hemolymph tissues where VCVs accrue [16], highlighting the chronic nature of AV disease.

### 5.2. AV Virions Contain Host-Coded Proteins

During virogenesis, many viruses sequester host proteins that become integral or associated components of their virions. Structural and scaffolding proteins, such as nuclear-derived histones, are essential for nucleic acid condensation and encapsidation in viruses like papillomavirus. Additionally, host-derived non-structural proteins, including enzymes, can play critical roles in viral attachment and entry, genome replication, egress, and immune evasion. These host–virus interactions are well-characterized in vertebrate-infecting viruses, particularly human pathogens [115,116,117,118,119,120,121,122,123].

Similar associations have been observed in insect viruses. For instance, baculoviruses incorporate between 11 and 101 host proteins, while the HvAV-3h virion can be purified with up to 292 heterologous proteins depending on the lepidopteran host infected [7,124,125]. Although it remains unclear whether many of these host proteins are passively incorporated or selectively packaged in virions, their known or presumed functions suggest potential roles in AV biology. A recent study [126] showed that HvAV-3h virions propagated in different insect larvae consistently contained 29 virus-encoded structural proteins, along with various host proteins. These include heat shock proteins (Hsp 27.2, Hsp 60, Hsp 70, Hsp 83), prophenoloxidases (PPO-1, PPO-2), V-type proton pump, epoxide hydrolase, carboxylesterase (CarE), and cytochrome P450. Catalase and glutathione S-transferase (GST) were also identified; these enzymes help mitigate oxidative stress by degrading hydrogen peroxide and neutralizing reactive intermediates, respectively. The protective functions are likely advantageous during early virogenesis [127]. Although the presence of these proteins varied across hosts, the study suggests that HvAV-3h may adapt its virion composition to counter host-specific pressures. Selected host proteins with potential roles in AV biology are discussed below.

#### Heat Shock Proteins (HSPs), Prophenoloxidases (PPOs), and Epoxide Hydrolase (EH)

HSPs play pivotal roles in viral infections by ensuring proper folding and stabilization of viral proteins, suppressing apoptosis, and remodeling the actin cytoskeleton [128,129,130,131,132,133,134,135]. For instance, HSP27 functions as a molecular chaperone, guiding viral proteins to achieve conformations essential for replication [134,135]. It also inhibits apoptosis, prolonging the survival of infected cells, and promotes actin polymerization, which supports cellular integrity and facilitates viral dissemination. HSP60 contributes to the assembly of viral replication complexes and regulates mitophagy, thereby modulating mitochondrial dynamics, bioenergetics, and viral replication.

HSP70 aids in folding viral proteins and helps viruses evade host immune defenses [134]. HSP83, the *Drosophila* ortholog of HSP90, enhances viral gene expression and facilitates viral entry into host cells. Similarly, HSP90 ensures the correct folding and stabilization of viral proteins, enabling their functional activity during replication [134].

It is paradoxical that HvAV-3h virions contain PPO-1 and PPO-2, proenzymes central to the insect immune system and the melanization response, which is vital not only for wound healing and development, but also for neutralizing pathogens, including viruses [136,137]. Upon activation, PPOs are converted into phenoloxidases (POs), which catalyze melanin production, forming a physical barrier around invading pathogens. Some viruses have evolved mechanisms to suppress PPO activity, thereby reducing melanization and evading immune encapsulation and destruction. Given their protective role against pathogens, the inclusion of PPOs in HvAV-3h virions seems counterintuitive. However, it is important to note that only the inactive proenzyme forms are packaged. Nevertheless, upon viral entry, their activation may trigger the production of reactive oxygen and nitrogen species and other oxidative intermediates [138] which could contribute to regulated cell death (RCD) pathways and VCV biogenesis, key processes in AV pathobiology.

EHs play a multifaceted role in viral infections by modulating lipid signaling molecules involved in inflammation and immune responses [139,140,141,142,143,144]. Viruses can alter EH activity to suppress host immunity, facilitating immune evasion. EHs also regulate key cellular signaling pathways by converting epoxides to diols, processes that viruses may hijack to enhance replication. Changes in EH activity can influence cell proliferation and apoptosis, both of which are commonly manipulated by viruses to create a more permissive environment for replication. Additionally, EHs are vital for detoxifying harmful epoxides.

Interestingly, insect juvenile hormone epoxide hydrolase (JHEH) shares structural and functional similarities with microsomal EHs and is evolutionarily related to haloalkane dehalogenases [145]. Both enzyme types hydrolyze epoxides into diols, and generic EHs may theoretically degrade juvenile hormone (JH) [140,146,147]. Since JH is essential for larval growth and development, its reduction can result in fewer molts and stunted larvae. AVs similarly disrupt host molecular signaling, leading to reduced feeding and abnormal development [51,148]. Although a direct link between AV infection and JH levels remains unconfirmed, AVs may indirectly influence hormonal regulation, contributing to the developmental defects observed in infected larvae.

## 6. A Unique Cytopathology Evolved in AVs for Dissemination Efficiency

Viruses disrupt host cell functions early in infection by triggering extrinsic apoptosis, ER stress, interferon responses, and immune evasion [110,149,150,151,152]. Viral components often activate cell surface receptors, initiating signaling cascades that lead to programmed cell death, which is frequently exploited to enhance replication and spread. Later, viruses may evade immune detection by cloaking themselves in apoptotic bodies (1–5 µm) or exosomes (30–150 nm) [153,154]. Viruses also induce cytopathic effects (CPEs), many of which are easily discernible by microscopy. Examples include nuclear shrinkage (herpesviruses), syncytia formation (paramyxoviruses, coronaviruses), Negri bodies (rabies virus), and cytoplasmic vacuoles (polyomavirus, papillomavirus) [155,156,157,158]. Insect viruses like NPVs form nuclear occlusion bodies composed of polyhedrin [7,159]. In contrast, AVs uniquely initiate cell death pathways to repurpose apoptotic vesicles into VCVs that support virogenesis and transmission [12,13,51,126].

Early ultrastructural studies by Federici [15] revealed that AV replication initiates in the nucleus, a trait shared by many dsDNA viruses [160], though notable exceptions exist, including poxviruses that replicate in the cytoplasm [161]. Unlike other nuclear-replicating dsDNA viruses that form replication centers to shield viral processes from host antiviral defenses [162], AVs diverge significantly. These replication centers, common in DNA viruses, reorganize nuclear architecture without relying on membranous structures like the replication organelles of RNA viruses [163]. Instead, they restructure chromatin and nuclear domains, including Promyelocytic Leukemia Nuclear (PML) bodies, Cajal bodies, interchromatin granules, and the nucleolus, to create specialized compartments for virogenesis [162,164,165,166].

In contrast to these DNA viruses, AVs manipulate both nuclear and plasma membranes, marking a unique cytopathological strategy to generate biochemically active VCVs [15,17]. This process is consistent across AV hosts and tissues. Early signs include nuclear enlargement and fragmentation, with infected cells expanding 5–10×, far more than the 2–3× seen in cytomegalovirus infections [167]. Plasma membrane invagination forms mitochondrial-lined cleavage planes where lipid vesicles emerge, varying by species: in SfAV-1a and TnAV-2, they extend inward from the periphery; in others, they initiate near the edge [16]. Fusion of these membranes splits the cell into 20–50 vesicles (~2–12 μm) that are released into the hemolymph within 2–3 days, reaching concentration of >10^8^ particles/mL by day 3–4 (Figure 1B,C) [15]. The acellular VCVs remain viable in the hemolymph throughout the larval stage and act as a source of infectious particles for mechanical transmission to caterpillars by endoparasitoid wasps.

### SfAV-1a VCVs Are Modified Bodies Derived from Regulated Cell Death Pathways

SfAV-1a induces a cytopathology that resembles apoptosis in the initial phase of VCV formation [1,12,15]. The process is similar to the biphasic cell death seen in HvAV-3h, in which initial pyroptosis-like features likely suppress host defenses, followed by apoptosis to aid viral packaging and dissemination (Figure 1D) [13,126]. However, SfAV-1a VCVs differ fundamentally from apoptotic bodies. Apoptosis typically involves cell shrinkage, chromatin condensation, nuclear fragmentation, membrane blebbing, DNA fragmentation, and formation of apoptotic bodies cleared by the innate immune system [168]. Although SfAV-1a activates apoptosis in vivo and in vitro, evidenced by membrane blebbing and nucleosomal DNA fragmentation (Figure 1F–H) [12], two key distinctions exist: (1) infected cells enlarge 5–10×, and (2) VCVs persist in tissue and hemolymph without degradation [1,2]

In vertebrates, apoptotic bodies are cleared via efferocytosis, guided by “find me” signals (lysophospholipids, lysophosphatidylcholine, sphingosine-1-phosphate, ATP, AMP, UTP) and “eat me” signals (phosphatidylserine) [169,170], and are then degraded by lysosomal hydrolases [171,172,173]. Despite apoptosis induction by SfAV-1a, VCVs evade clearance by lepidopteran hemocytes and histiocytes [174,175,176,177,178,179,180]. The lipid makeup of AV VCV membranes is unknown but likely lacks typical clearance signals. Alternatively, if these signals are present, the immune system may be overwhelmed by rapid tissue degradation and VCV proliferation [15]. Unlike viruses such as SARS-CoV-2 or ASFV, which exploit efferocytosis for immune evasion and transmission [170,181,182,183,184,185], AVs do not use this pathway as a “Trojan horse.” Instead, they reprogram apoptotic bodies into stable subcellular compartments that enhance viral replication without mediating intercellular spread within histiocytes and hemocytes.

## 7. AV Cytopathology

As mentioned earlier, the structural and pathobiological characteristics of AVs are well documented, though the functional molecular biology is in its early stages [1,2]. Nonetheless, recent comparative genomic analyses of several AVs (Table 1) along with in vivo transcriptomes [16,17,18,19,186] and functions of a few AV-coded proteins are aiding the development of theoretical models to elucidate these cytopathological features. In this context, we present theoretical molecular models to explain AV cytopathology leading to the generation of VCVs, primarily based on shared proteins encoded by these viruses (Table 3) and their putative associations with characterized cellular pathways.

### 7.1. Cellular Invasion

The mechanisms by which AV invade cells remain unclear, but insights from NCLDV members suggest possible strategies such as membrane fusion, cell–cell fusion, and various forms of endocytosis, including phagocytosis, pinocytosis, clathrin-mediated, and caveolar/raft receptor-mediated pathways [187]. Unlike most NCLDVs, which possess envelope protrusions or fibers that aid in these processes, AV virions lack such structures [187]. Given the diversity in AV tissue and cellular tropism, infection likely depends on specific ligand-receptor interactions, particularly for VCV-free virions. Alternatively, host cell engagement may involve distinct ligands on the VCV membrane or direct membrane fusion, though the latter is improbable due to the large size of the VCVs.

Pathogens often initiate cell entry by manipulating cytoskeletal proteins, particularly surface fiber, a strategy well-documented in Vaccinia virus, a poxvirus within the NCLDV group [188,189,190]. For AVs, only one in vitro study has examined actin’s role in infection [191]. Treatment of *Heliothis zea* fat body (HzFB) cells with cytochalasin D did not block HvAV-3e replication, suggesting actin is not essential for viral entry. However, the disruption inhibited membrane blebbing, vesiculation, and cell cleavage, processes critical for VCV formation, highlighting the importance of cytoskeletal reorganization in AV-induced pathology.

### 7.2. Oxidative Stress Suppression

Recent studies have elucidated the role of the Nrf2/ARE antioxidant pathway in HvAV-3h-infected *S. exigua* larvae [192,193]. Upon infection, larvae exhibit an early oxidative stress response characterized by increased activity of antioxidant enzymes such as superoxide dismutase (SOD) and peroxidase (POD), alongside elevated expression of NADPH oxidase (SeNOX) and peroxidase genes (SePOD). The response activates SeNrf2 and SeMaf, key regulators of the Nrf2/ARE pathway. Functional analyses using RNA interference showed that silencing SeNrf2 significantly reduced SePOD expression, enhanced viral replication, and increased larval mortality, indicating a protective role for Nrf2 in antiviral defense. Furthermore, the knockdown of SeNOX suppressed both SeNrf2 and SePOD expression suggesting that ROS production via SeNOX is upstream of Nrf2 activation. These findings highlight a critical regulatory axis involving SeNOX, Nrf2, and antioxidant enzymes that support host cell survival during viral infection.

### 7.3. Nucleic Acid Metabolism

AVs encode proteins that manipulate host nucleic acid metabolism to support viral replication and gene expression. Transcriptomic data show many of these genes are expressed early during infection [16,17,18]. AVs replicate their DNA using B family polymerases similar to eukaryotic α, δ, and ε polymerases, featuring proofreading exonuclease domains [194,195]. Unlike other large DNA viruses, AVs lack a PCNA-like clamp protein [196,197,198], suggesting reliance on host factors for replication fidelity. D5-like helicase/primase proteins, conserved across NCLDVs and bacteriophages, unwind DNA during replication [81,199,200,201,202]. To support replication and repair, AVs encode thymidine kinases [203,204], topoisomerase I [205], DNA ligase [206], and various ATPases, helicases, and nucleases including S1/P1 [100,200,207,208,209,210,211,212,213]. FEN-1/FLAP-like nucleases assist in RNA primer removal [214], and G5R orthologs may regulate replication and repair [81,215,216]. These enzymes are conserved among NCLDVs [28,196].

AVs also modulate RNA metabolism via DNA-dependent RNA polymerase subunit C, RNA polymerase II subunits, and transcription factors like TFIIF, VLTF2, and Yabby-like proteins [217]. Yabby-like TFs, with zinc finger and HMG-like domains, regulate gene expression through chromatin interactions [218,219]. FEN-1/FLAP-like nucleases contain XPG N-I motifs resembling VHS (virus-induced host protein shutoff) that degrade host mRNAs to favor viral expression [220,221,222]. All AVs encode RNase III-like homologs with Dicer-like domains that cleave dsRNA and regulate gene expression [223,224,225,226]. These enzymes support RNA maturation, decay, and silencing, including antiviral defense and mitochondrial regulation [227,228,229]. Though rare in animal dsDNA viruses, they occur in Paramecium bursaria chlorella virus 1 and Rock bream iridovirus [229,230,231], and in plant viruses like Sweet potato chlorotic stunt virus, where they suppress RNA silencing [232]. In HvAV-3e, a capsid-derived microRNA targets DNA polymerase transcripts [233], and its RNase III suppresses host RNA interference, marking the first known use of RNase III by an insect virus to evade silencing [234].

### 7.4. Delaying Immune and Cell Death Pathways—Antiapoptotic Proteins

As mentioned above, the hallmark of AV cytopathology is the regulated cascade leading to bioactive VCV formation, requiring delayed activation of pyroptosis and apoptosis [12,13,125]. This delay enables early virus-directed programs to initiate nuclear disintegration, cellular expansion, and cleavage. Host cells typically trigger apoptosis, necroptosis, or pyroptosis to restrict viral spread [14,39,90,180,235,236], while viruses counter with anti-apoptotic proteins such as serpins (e.g., CrmA), P35 family proteins (e.g., P35, P49), FLIPs (e.g., vFLIP), and IAPs (e.g., Op-IAP, AMV-IAP, A224L), which inhibit caspases and extend cell survival [124,237,238]. IAPs, defined by BIR domains and often RING domains, ubiquitinate executioner caspases-3, -7, and sometimes initiator caspase-9 (intrinsic pathway), but not caspase-8 (extrinsic pathway), targeting them for degradation [239,240,241]. Although AVs lack serpin, P35, and FLIP orthologs, they encode multiple IAP-like proteins with degenerate BIR and E3 ligase-like domains. While not orthologous to OpIAP3 or CpIAP [242,243,244], AVs such as HvAV-3e-j, TnAV-6a, and SfAV-1a encode 5, 2, and 4 IAPs/IAP-like proteins, respectively, with at least one incorporated into virions [45,68,245].

In SfAV-1a, *lap*-like genes are expressed early [18], likely suppressing intrinsic apoptosis via caspase inhibition. SfAV-1a also encodes a unique viral executioner caspase expressed late in infection [12,18], suggesting early anti-apoptotic signaling is redirected for VCV production. HvAV-3h may use IAPs and pyroptosis together to delay apoptosis [13,125], with its six IAP homologs potentially inactivating both initiator and executioner caspases. In SfAV-1a, early suppression may be reversed by its own caspase, facilitating rapid cleavage and VCV formation. TnAV and HvAV encode caspase-like proteins but lack executioner caspases. While IAPs inhibit caspases, they do not directly block pyroptosis, which involves inflammasome activation, gasdermin-mediated pore formation, and cytokine (IL-1β, IL-18) release [246,247,248]. IAPs like cIAP1, cIAP2, and XIAP modulate pyroptosis via ubiquitination and regulation of NF-κB and RIPK1 signaling. NAIP (BIRC1), an atypical IAP, senses microbial ligands and activates the NLRC4 inflammasome, triggering caspase-1 and pyroptosis. Thus, IAPs influence cell fate contextually, depending on stimuli and environment.

Beyond cell death, IAPs regulate cell cycle, DNA repair, immune signaling, and protein degradation [249,250,251,252]. As only one IAP is found in AV virions, the others may serve expanded roles. For instance, HvAV-3e ORF028, with an imperfect BIR domain and an intact RING domain, failed to block chemically induced apoptosis, implying other viral genes contribute to anti-apoptotic activity [233]. Additionally, SfAV-1a encodes a virokine, Diedel, originally discovered in *Drosophila melanogaster* [253], which inhibits the Imd-NF-κB pathway and promotes epithelial survival. This immunosuppressive virokine (ORF121), the only known insect virus-coded virokine, is highly expressed in SfAV-1a-infected *S. frugiperda* larvae, suggesting a role in immune evasion [18].

### 7.5. Cellular Cleavage and Formation of VCVs

VCV biogenesis is driven by AV-regulated processes including programmed cell death, viral gene expression, genome replication, cytoskeletal remodeling, and membrane synthesis. These events maintain VCVs in a viable state essential for virogenesis. Unlike other viruses, AV transcription occurs primarily within VCVs rather than host tissues [17]. Transcriptomic analyses show a temporal correlation between host cytoskeletal gene expression and AV/mitochondrial genes in VCVs from SfAV-1a and TnAV-6a infected hemolymph [16]. Genes encoding actins (21), tubulins (29), dyneins (21), and kinesins (13) are significantly upregulated ~48 h post-infection, coinciding with VCV maturation. These proteins likely support hypertrophy, organelle motility, and membrane remodeling.

In SfAV-1a, apoptosis begins at approximately 9 h post-infection in SF21 cells and around 48 h in larval fat body tissue, with VCVs appearing in the hemolymph by 72 h (Figure 1C). In SF21 cells, the caspase inhibitor z-DEVD-fmk completely blocks the apoptotic response, demonstrating the essential role of executioner caspases in the virus’s life cycle [12]. As chromosomal DNA fragments during apoptosis, nuclear gene transcription likely contributes minimally to VCV formation, although host mRNA translation may persist and support early viral protein synthesis. The timing and coordination of these events suggest that SfAV-1a leverages host apoptotic machinery to facilitate vesicle morphogenesis. Conserved AV proteomes [16,17,18,19] further support the hypothesis that VCV development is orchestrated through a complex interplay of regulated cell death, lipid metabolic reprogramming, and vesicle biogenesis, underscoring the virus’s sophisticated manipulation of host cellular architecture.

### 7.6. Proapoptotic Proteins of AVs

Apoptosis, a well-characterized form of programmed cell death in both invertebrates and vertebrates [254,255,256,257], is mediated by procaspases [241,258] that, once activated, drive chromatin condensation, nucleosomal fragmentation, membrane blebbing, and cytoskeletal reorganization [256,259], all features of SfAV-1a-induced cytopathology [12,15]. Apoptosis proceeds via extrinsic (plasma membrane death receptor-mediated activation of caspase-8/-10) or intrinsic (mitochondrial permeabilization and cytochrome c release activating caspase-9) pathways, both converging on activation of executioner caspases (caspase-3, -6, -7) [260,261]. In AVs, the intrinsic pathway is more relevant, as AVs encode mitochondrial-destabilizing enzymes including caspase-like proteins, cathepsin B, and lipid-metabolism-related enzymes [2,18].

SfAV-1a encodes a functional executioner caspase (ORF073) with conserved P20/P10 domains, a QSCLG catalytic motif [12], and an IAP-binding motif similar to the motif in caspase-9 [262,263,264]. This caspase auto cleaves in *E. coli*, cleaves z-DEVD-aminoluciferin, induces apoptosis independently of external stimuli, and its silencing reduces apoptotic bodies and VCV formation [12]. Other AVs lack functional executioner caspases. HvAV-3e encodes HvAV-Casp (ORF165) that shares moderate identity with SfAV-1a and human caspase-7, but lacks catalytic residues and autocleavage sites, and does not induce apoptosis [65]. Its silencing reduces MCP levels, suggesting a role in virion assembly. TnAV-6a encodes TnAV-Casp2 (ORF072) that is homologous to caspase-2, but the protein lacks a CARD domain and canonical catalytic residues [259,265]. Thus, SfAV-1a is unique among all known viruses in using a virus-coded executioner caspase to drive cytopathology [12]. Beyond apoptosis, viruses often exploit caspases to regulate viral protein function, aiding trafficking, transcription, virion maturation, and egress, as seen in the cellular biology induced by influenza, hepatitis C, HPV, and astrovirus [237,266,267,268,269]. As such, AV caspases may serve similar multifunctional roles.

Cathepsin B, a lysosomal cysteine protease [270], is present in AVs as well as other large double-stranded DNA viruses, including iridoviruses (IIVs) and baculoviruses [271,272]. While the role of cathepsin B in IIV6 remains unclear, baculoviral cathepsin L, along with chitinase, is known to contribute to larval tissue degradation and liquefaction during infection [272,273]. In contrast, AV-infected larvae exhibit minimal tissue breakdown, suggesting that cathepsin activity is tightly regulated. Nevertheless, under cellular stress, lysosomal membrane permeabilization can release cathepsin B into the cytosol, where it participates in multiple cell death pathways, including apoptosis, pyroptosis, necroptosis, autophagy, and ferroptosis [274,275,276,277,278]. In apoptosis, cathepsin B cleaves Bid to tBid, activating Bax/Bak and mitochondrial outer membrane permeabilization (MOMP), likely triggering activation of the SfAV-1a caspase [278]. Whether or not AV cathepsins are trafficked into the lysosome is unknown, but if it remains in the cytoplasm after synthesis, it may accelerate activation of proapoptotic pathways and cellular degradation. Nonetheless, unlike baculovirus infections, AVs likely suppress proteolytic degradation to prolong larval viability. In this regard, it was recently shown that HvAV-3h ORF31 suppresses cathepsin and chitinase activity in *S. exigua* larvae [279], and the presence of homologs in SfAV-1a (ORF32) and TnAV-6a (ORF26) suggests a conserved mechanism supporting persistent infection.

### 7.7. Lipid Metabolism, Ceramide-Sphingosine-1-Phosphate Rheostat, and VCV Formation

**Figure 2:**
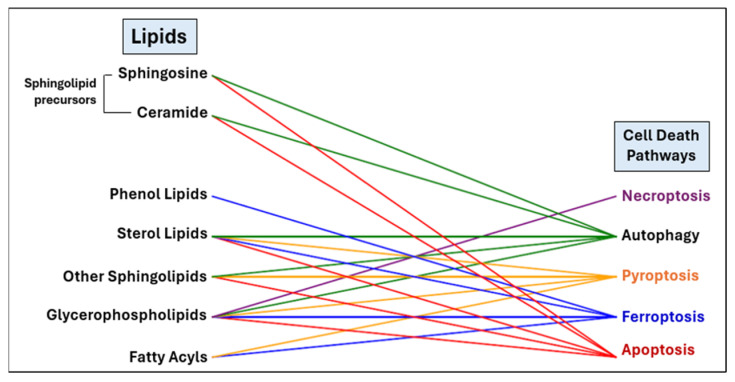
Lipid classes and associated cell death pathways. This schematic summarizes the involvement of major lipid classes in regulated cell death (RCD) pathways. Color-coded lines indicate the lipid-pathway associations based on experimental and mechanistic evidence. This simplified display is based on descriptions by Lin, Z. et al. [280].

**Figure 3:**
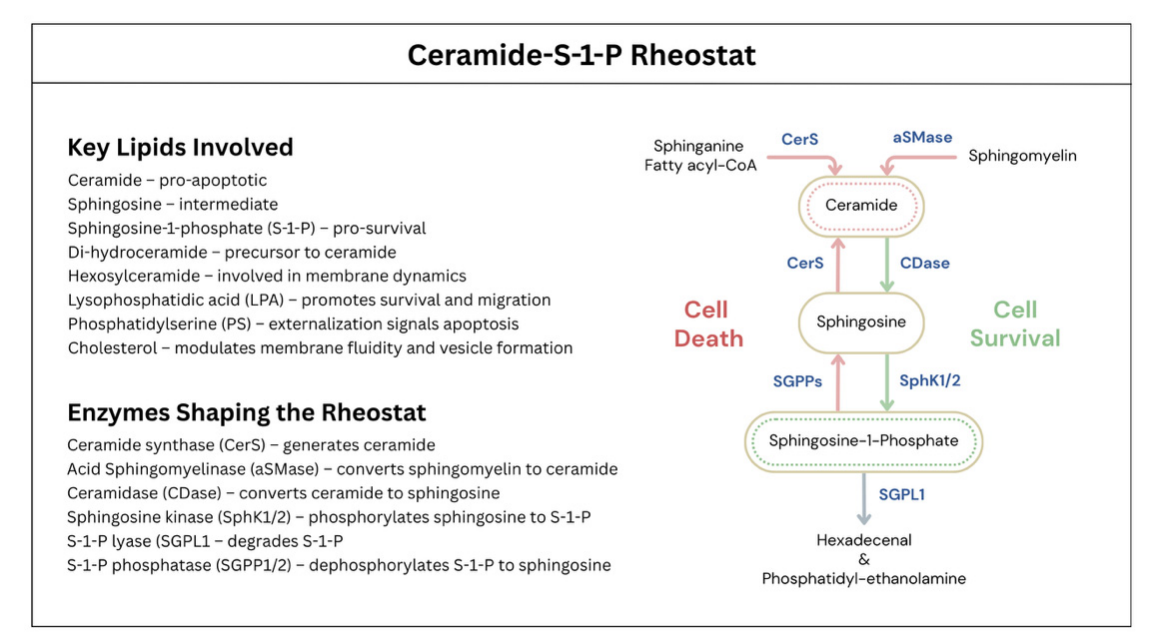
The ceramide–sphingosine-1-phosphate (S-1-P) rheostat and its role in regulating cell death and survival. The rheostat represents a dynamic balance between pro-apoptotic and pro-survival sphingolipids. Ceramide, generated via ceramide synthase (CerS) or sphingomyelinase (SMase), promotes apoptosis and membrane remodeling conducive to vesicle formation, a hallmark of AV cytopathology. Ceramide can be converted to sphingosine by ceramidase (CDase) and subsequently phosphorylated by sphingosine kinase (SphK1/2) to form S-1-P, a lipid that supports cell survival, proliferation, and inflammatory signaling. S-1-P levels are regulated by S-1-P lyase (SGPL1), which irreversibly degrades it to hexadecenal and phosphatidylethanolamine, and S-1-P phosphatases (SGPP1/2), which revert it to sphingosine + inorganic phosphate. Additional lipids involved include dihydroceramide (a biosynthetic precursor), hexosylceramide (involved in membrane trafficking), lysophosphatidic acid (promote survival and migration), phosphatidylserine (externalized during apoptosis), and cholesterol (modulate membrane fluidity and vesicle biogenesis). Together, these lipids and enzymes may orchestrate the cellular response to AV infection, influencing both cell death and the generation of bioactive acellular virion-containing vesicles in which virogenesis is completed.

Over the past two decades, lipid metabolism has emerged as a critical regulator of cell death and survival during viral infections (Figure 2 and Figure 3), with many viruses exploiting host lipid biosynthetic pathways to support replication [280,281]. Upregulation of fatty acid and complex lipid synthesis provides structural components for viral envelopes and replication organelles [282]. DNA viruses like cytomegalovirus, Epstein–Barr virus (EBV), and Kaposi’s sarcoma-associated herpes virus (HHV8) activate host enzymes such as fatty acid synthase (FASN), while RNA viruses like HCV and dengue virus remodel intracellular membranes to enrich replication complexes with phospholipids and cholesterol. To sustain replication, viruses manipulate host cell death pathways, often targeting the ceramide-sphingosine-1-phosphate (S-1-P) rheostat, a key axis in sphingolipid metabolism that governs cell fate under stress [283,284].

Ceramide and sphingosine promote apoptosis by inducing mitochondrial dysfunction and lysosomal destabilization, which activate pro-apoptotic Bcl-2 proteins and trigger mitochondrial outer membrane permeabilization (MOMP). On the other hand, S-1-P, generated by sphingosine kinases (SphK1/2), supports cell survival and enhances viral replication. It does so by activating S-1-P receptors and downstream signaling pathways that suppress apoptosis and help restore mitochondrial integrity. Viral infections activate host signaling cascades (ERK/MAPK, JAK/STAT, Akt/mTOR), which phosphorylate SphK1 and elevate S-1-P levels, inhibiting Bax and reinforcing survival signaling [283,284]. Viruses such as influenza A, HIV-1, herpesviruses, and SARS-CoV-2 exploit the lipid rheostat to prolong host cell viability and replication [285,286,287,288,289]. Given AVs’ known manipulation of apoptosis and pyroptosis [1,2,12,13], they likely exploit the rheostat to optimize VCV biogenesis. Viruses also reprogram host lipid metabolism via the PI3K/Akt/mTOR pathway, which also occurs in insects [290], and regulators like SREBPs, FASN, and ACC1 [291,292,293,294], rerouting carbon sources such as citrate (via ATP-citrate lyase) to generate acetyl-CoA for fatty acid and cholesterol synthesis. These lipids are essential for membrane biogenesis, lipid rafts, and replication compartments.

In AVs, mitochondrial localization along lipid cleavage planes during VCV formation [15] likely supports ATP production and supplies precursor molecules for lipid biosynthesis. Following AV infection, cellular hypertrophy and the formation of up to 50 VCVs per cell [16] depend on de novo lipid synthesis to expand membranes. Early virogenesis may rely on host-directed lipid production [295,296], but as cell death programs activate, AV-coded enzymes likely supplement or replace host biosynthetic machineries. This shift is crucial, as apoptosis typically disrupts host metabolism, including lipid synthesis, which is essential for VCV formation. AVs encode a unique set of enzymes involved in lipid metabolism, including phosphate acyltransferases (PATs), fatty acid elongases (FA-ELOs), esterase/lipase, and patatin-like phospholipases (PNPLA-2). These enzymes not only generate lipid precursors but also produce intermediates like ceramides and lysophospholipids that may modulate cell death programs (Figure 2) [297,298,299].

Phosphate acyltransferases (PATs) are members of the PlsC family [300] that include GPATs and AGPATs which are essential for glycerophospholipid biosynthesis [301]. These enzymes generate lysophosphatidic acid and phosphatidic acid, key intermediates sequestered by viruses for envelope formation, replication organelles, and immune evasion via altered membrane dynamics [301].

HvAV-3e ORF19 is the only AV esterase/lipase homolog studied to date. Although its enzymatic activity was not confirmed, it is essential for viral replication and cell cleavage [302]. Similar roles are seen in other viruses: Vaccinia virus p37 enhances infectivity by converting intracellular virions into extracellular forms [303,304], while Marek’s disease virus vLIP, though catalytically inactive, retains a serine nucleophile critical for replication, likely functioning in fatty acid binding [305].

Fatty acid elongases (FA-ELOs), part of the GNS1/SUR4 family [306], catalyze the rate-limiting step in ‘very long chain fatty acid’ (VLCFA) synthesis, producing precursors for ceramides, sphingolipids, and phospholipids that shape membrane properties [307]. In *S. cerevisiae*, ELO3 modulates plasma membrane H^+^-ATPase activity via lipid dynamics [308,309]. VLCFAs are linked to apoptosis and immune regulation, and viruses such as hepatoviruses, flaviviruses, and coronaviruses exploit these pathways to remodel cellular membranes and evade immune responses [310,311]. Transcriptomic and metabolomic data reveal dynamic regulation of cholesterol and VLCFA synthesis during viral stress, indicating viral manipulation of these host metabolic processes [307].

The PNPLA family, comprising nine lipid-metabolizing enzymes, is activated by oxidative stress and implicated in membrane homeostasis, signaling, and cell death [312,313,314]. Patatin, a lipid acyl hydrolase from *Solanum tuberosum*, inhibits *Diabrotica* larval growth via its enzymatic activity [315,316]. In *Rickettsia parkeri*, an obligate intracellular bacterium, a patatin-like phospholipase A2 (PNPLA) known as Pat1 facilitates vacuolar escape and immune evasion by preventing recognition by host galectins and autophagy receptors such as p62. This activity enables cytosolic access and actin-based motility, promoting cell-to-cell spread. Mutants lacking Pat1 show reduced virulence, underscoring its essential role in pathogenesis [317]. These findings suggest AV PNPLAs may similarly mediate mitochondrial-lysosomal interactions, intercellular spread, and immune evasion.

### 7.8. Role of Ascovirus Lipid Enzymes in Programmed Cell Death Pathways

**Figure 4:**
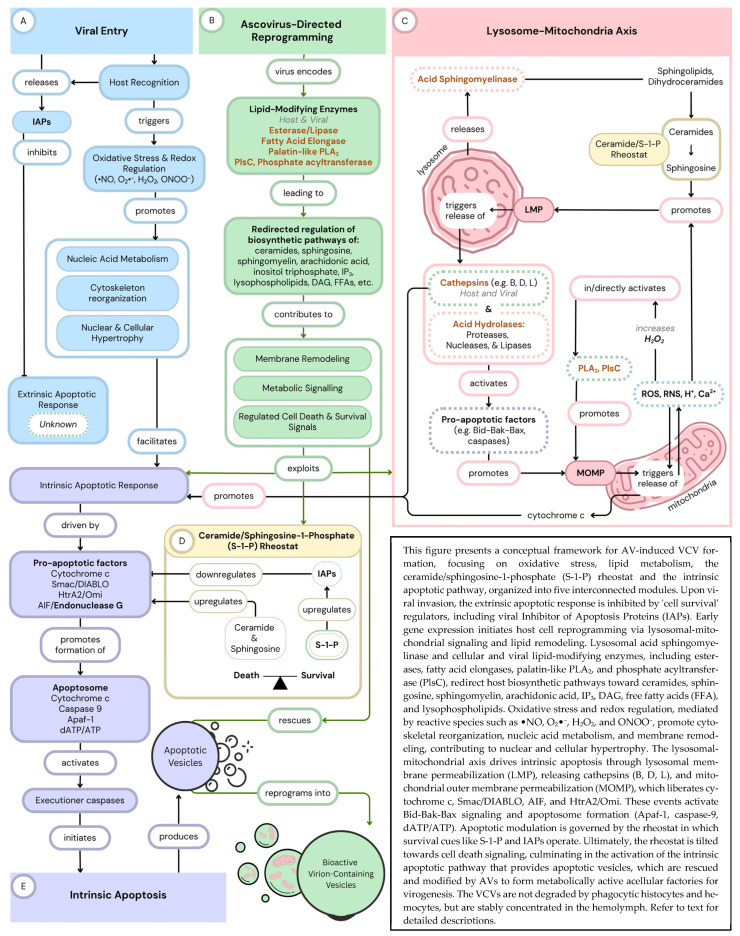
Flow-chart model of pathways involved in the production of ascovirus (AV) virion-containing vesicles (VCVs).

The predicted activities of AV lipid-modifying enzymes suggest functions beyond membrane biogenesis, including roles in virion and VCV formation. Enzymes such as FA-ELO and PNPLA-2 may contribute to sphingolipid biosynthesis, potentially influencing cell death pathways, as illustrated in Figure 2 and Figure 4, Table 4. Overexpression of the patatin-related enzyme pPLAIIIβ in *Arabidopsis thaliana* increases levels of sphingolipid precursors, including 3-ketosphinganine, along with long-chain bases, ceramides, complex sphingolipids (e.g., sphingomyelin, glycosphingolipids), and arachidonic acid [318]. These lipids are bioactive molecules known to regulate apoptosis, necroptosis, pyroptosis, ferroptosis, autophagy, and inflammation [280,319,320,321,322], highlighting potential links between AV lipid metabolism, virogenesis, and host cell death responses as modeled in Figure 4.

Ceramide, a central mediator of stress and apoptosis [323,324], accumulates via de novo synthesis [325,326], sphingomyelin hydrolysis [327,328], inhibition of ceramide breakdown, or altered glucosylceramide metabolism [329]. Hydrolysis-induced ceramide triggers rapid apoptosis, while de novo synthesis has delayed effects [330]. Ceramide activates lysosomal cathepsins, modulates Bcl-2 proteins, promotes Bax oligomerization, and disrupts mitochondrial function, leading to cytochrome c release and intrinsic apoptosis [331,332,333,334,335,336]. Sphingosine, derived from ceramide, also promotes apoptosis by inhibiting survival pathways (PKC, Akt, PI3K-Akt-mTOR) [337,338], activating Bid and caspases [339,340,341], and destabilizing lysosomal and mitochondrial membranes [275,340,342]. Arachidonic acid, released by PNPLA2-mediated phospholipid hydrolysis [343], enhances cytochrome c release and caspase activation [344,345,346], and may stimulate ceramide synthesis via sphingomyelinase [347]. Overexpression of iPLA2 amplifies ER stress-induced apoptosis [348].

Reactive oxygen species, particularly hydrogen peroxide (H_2_O_2_), further modulate lipid signaling and cell death [349]. While H_2_O_2_ does not directly activate PlsC, it induces bioactive lipids like HHP-C9 (1-hydroxy-1-hydroperoxynonane) through membrane oxidation [350,351], and triggers second messenger production (DAG, IP_3_). It also directly activates cytosolic PLA_2_ via phosphorylation (PKC, MAPK), lipid peroxidation, and calcium signaling, contributing to cellular instability and influencing the ceramide/sphingosine–S-1-P rheostat [349].

## 8. Lysosome–Mitochondria Feedback Loop and Intrinsic Apoptotic Induction

The theoretical model for AV VCV biogenesis (Figure 4) centers on the lysosomal–mitochondrial axis, a key regulatory hub of programmed cell death. During AV infection, this axis is not only activated but repurposed to support vesicle-based replication by providing structural components and metabolic substrates.

Lysosomal membrane permeabilization and mitochondrial dysfunction may act in concert to initiate cell death signaling, while simultaneously facilitating viral egress and replication. This dual role underscores the complexity of host–virus interactions and the potential of AVs to manipulate conserved cellular pathways for their own propagation. The interplay between lysosomal and mitochondrial pathways forms a tightly regulated feedback loop that governs intrinsic apoptosis [352,353]. In the model, SfAV-1a executioner caspase, cathepsin B, and lipid-modifying enzymes are central mediators. Lysosomal membrane permeabilization (LMP), triggered by oxidative stress during infection, releases cathepsin B and acid hydrolases into the cytosol, activating pro-apoptotic proteins such as Bid, Bad, and Bax. This promotes MOMP, cytochrome c release, and apoptosome formation (Apaf-1/cytochrome c/caspase-9), leading to activation of executioner caspases, including the SfAV-1a caspase [354]. LMP also releases acid sphingomyelinase (aSMase), which hydrolyzes sphingomyelin into ceramide. Ceramide integrates into mitochondrial membranes, enhances pore formation, and amplifies MOMP and cytochrome c release, reinforcing the apoptotic loop and contributing to organelle clearance via mitophagy [355,356].

Beyond its canonical role in apoptosis, aSMase may play a pivotal role in virion-containing vesicle (VCV) biogenesis during AV infection. aSMase catalyzes the hydrolysis of sphingomyelin, the most abundant sphingolipid in eukaryotic membranes, into ceramide and phosphorylcholine, initiating membrane remodeling. Ceramide’s biophysical properties, particularly its conical shape, promote negative membrane curvature, which facilitates inward budding, membrane invagination, and vesicle formation [357,358]. These processes extend to intracellular compartments, enhancing endocytosis and multivesicular body (MVB) development, both of which are critical for viral genome encapsidation and egress.

Importantly, pathogen-induced translocation of aSMase from lysosomes to the cytoplasm leads to the formation of ceramide-enriched microdomains at the plasma membrane and within endomembrane systems. This spatial redistribution of aSMase enables localized ceramide production, which may serve as a scaffold for VCV morphogenesis by recruiting specific viral and host factors [359]. Such translocation events have been implicated in viral entry and replication across multiple systems, suggesting that AVs may similarly exploit this mechanism.

Moreover, AVs could manipulate the ceramide–S-1-P rheostat, a key regulator of cell fate [283,284], to simultaneously trigger apoptosis and repurpose host membranes for vesiculogenesis. This dual strategy would allow AVs to convert apoptotic signaling into a structural platform for viral replication and dissemination, highlighting aSMase as a potential molecular switch in the transition from cell death to viral propagation.

To experimentally validate the hypothesis that aSMase contributes to VCV biogenesis in AVs, several complementary approaches can be employed. First, inhibition of aSMase using agents such as fumonisin B1, imipramine or desipramine can be used to assess whether blocking ceramide production disrupts vesicle formation and membrane remodeling during AV infection [360,361,362]. Indeed, our preliminary analyses show that fumonisin B1 and desipramine inhibit vesicle formation in SfAV-1a-infected SF21 cell. Second, lipidomic profiling of infected versus uninfected cells can quantify ceramide enrichment at VCV budding sites, providing biochemical evidence for sphingomyelin hydrolysis and ceramide accumulation [363]. Third, live-cell imaging using fluorescent ceramide analogs or genetically encoded ceramide-binding probes can be used to visualize ceramide microdomain formation and membrane curvature in real time [364]. Finally, genetic knockdown or CRISPR-mediated silencing [365,366] of host aSMase could help determine its necessity for VCV morphogenesis, with expected outcomes including impaired vesicle formation and reduced viral replication. Together, these experiments offer a robust framework to test whether AVs exploit aSMase-mediated ceramide remodeling not only for apoptosis but also for structural reprogramming of host membranes during viral replication.

### 8.1. Viral Exploitation of Mitophagy to Balance Apoptosis and Replication

Although mitophagy is well-characterized in mammalian viral systems [367,368], its role in AV infection remains hypothetical. Many viruses, including hepatitis B virus, hepatitis C virus, influenza A virus, and herpes simplex virus type 1, activate mitophagy via host proteins such as Parkin, PINK1, and FUNDC1 to suppress apoptosis and prolong cell survival [367,369]. This process preserves mitochondrial integrity, prevents cytochrome c release, and supports viral replication. In healthy cells, mitophagy maintains mitochondrial quality by removing damaged organelles, and during infection, it functions as part of the host’s regulated cell death response to limit viral spread [370]. In AVs, particularly SfAV-1a, transcriptomic data suggest a dual strategy: inducing limited mitochondrial dysfunction to initiate intrinsic apoptosis, while preserving active mitochondria for vesicle biogenesis. Zaghloul et al. [19] showed that mitochondrial genes such as ATP6, ATP8 synthase, and NADH dehydrogenase subunits were upregulated in AV-induced vesicles, indicating sustained mitochondrial activity. This suggests AVs may balance mitochondrial damage and preservation to create an intracellular environment where apoptosis scaffolds vesicle formation, and functional mitochondria support lipid synthesis, virogenesis, and VCV maturation.

To address the speculative nature of this model and make it experimentally testable, we propose several assays. First, live-cell imaging using mitochondrial membrane potential dyes, such as JC-1 or TMRE, can be used to assess mitochondrial integrity during AV infection. These dyes have been validated for detecting mitochondrial depolarization in various cell types [371]. Second, immunofluorescence or Western blotting for mitophagy markers, including PINK1, Parkin, and LC3-II, can help determine whether mitophagy is activated in AV-infected cells. These markers have been used to track mitophagy in macrophages under stress conditions [372]. Third, transmission electron microscopy (TEM) can be employed to visualize mitochondrial morphology and vesicle formation, as demonstrated in studies of respiratory syncytial virus-induced mitochondrial remodeling [373]. Finally, RNAi or CRISPR-mediated knockdown [365,366] of mitophagy-related genes such as Parkin or PINK1 could be used to assess their impact on VCV formation and viral replication. We note that gene silencing approaches have been successfully applied to study mitochondrial dynamics and development in *Arabidopsis* [374]. Together, these approaches would help validate whether AVs exploit mitophagy not only to initiate apoptosis but also to reprogram host membranes for vesiculogenesis.

### 8.2. Ceramide-Driven Feedback and Viral Reprogramming of Apoptosis

The lysosomal–mitochondrial feedback loop is amplified by ceramide, which promotes MOMP and lysosomal destabilization, triggering a cycle of protease release and organelle damage [367,368]. Caspase-3 intensifies this loop by cleaving lysosomal-associated membrane proteins (LAMPs), enhancing LMP and apoptotic signaling. Rather than allowing the completion of cell death to limit viral replication, AVs intercept this cascade by converting apoptotic bodies into metabolically active vesicles. These vesicles are likely enriched with specialized lipids, including long- and very-long-chain fatty acids, which stabilize membranes and enhance resistance to host defenses and environmental stress [375,376]. This reprogramming transforms intrinsic apoptosis from a host-protective mechanism into a platform for AV replication, enabling the formation of extracellular vesicle-like compartments that support viral propagation.

## 9. Factors Influencing the Persistence of AVs in Nature

To date, the impact of overwintering in parasitoid wasps and lepidopteran hosts on AV persistence remains unexplored. Nonetheless, several ecological and molecular features suggest that AVs possess considerable environmental resilience. These viruses produce abundant VCVs, which can remain infectious for extended periods within host hemolymph or perhaps external substrates. VCVs may persist in diapausing or late-instar larvae or pupae, or in environmental reservoirs such as soil and leaf litter, where cooler temperatures slow VCV and viral degradation. Additionally, overwintering parasitoid wasps may act as passive carriers, retaining viral particles on their ovipositors until spring emergence. Together, these mechanisms suggest that AVs are buffered against seasonal population declines and capable of reinitiating transmission as host and vector activity resumes. It is likely that the ubiquity of parasitoid wasps and lepidopteran larvae in terrestrial ecosystems provides a stable ecological niche that supports long-term viral persistence.

Beyond these strategies, a recently identified gene family known as the Parasitoid Killing Factor (PKF) further enhances ascovirus (AV) fitness by directly targeting competing parasitoid larvae within the host [377]. PKF genes encode a class of proteins that selectively induce apoptosis in developing parasitoid larvae, particularly those belonging to the subfamily *Microgastrinae*, which are common endoparasitoids of lepidopteran hosts. This targeted cytotoxicity effectively eliminates the parasitoid threat, thereby preserving host tissues for viral replication and prolonging host viability, both critical for successful AV transmission.

Molecular analyses suggest that PKF proteins act by disrupting mitochondrial integrity and activating caspase-dependent apoptotic pathways in the parasitoid cells, while sparing host tissues [377]. The specificity of this response implies a finely tuned evolutionary adaptation, likely shaped by intense selective pressure in virus-parasitoid-host interactions.

Remarkably, PKF genes are conserved across multiple viral taxa, including entomopoxviruses, AVs, and baculoviruses, indicating a shared defense strategy against parasitoids [377]. Their presence in both viral and insect host genomes points to historical horizontal gene transfer events and co-evolutionary dynamics. This interaction not only promotes viral persistence but, arguably, also shapes insect population dynamics, contributing to ecological balance.

## 10. Biocontrol Potential of Ascoviruses: Opportunities and Challenges

The unique pathology of AVs offers potential for sustained suppression of agricultural lepidopteran pests. However, AVs show limited effectiveness when ingested by insect hosts, as they are poorly infectious via the oral route. The reliance on wasps for mechanical transmission significantly restricts the feasibility of using AVs in large-scale field applications for pest control. Nevertheless, recent investigations of co-infection with *Bacillus thuringiensis* (Bt) have sought to overcome these limitations [378]. Strains of Bt are widely used globally to control larvae of lepidopteran pests of agriculture and nuisance and vector mosquitoes and blackflies that transmit viral and parasitic pathogens to humans and animals. The larvicidal activity of Bt is due primarily to Cry (crystal) and Cyt (cytotoxic) proteins that are synthesized and crystallized during sporulation of the bacterium, and which are solubilized and activated in the alkaline midgut where they destroy the midgut epithelia leading to larval death [282,379,380,381,382]. As Bt-derived Cry proteins disrupt the midgut epithelium, it is conceivable that Bt toxins could facilitate AV entry into the larval hemocoel, bypassing the need for parasitoid-mediated transmission.

Building on this concept, Yu et al. [378] tested formulations combining Bt subsp. kurstaki (Btk) with HvAV-3h and HvAV-3j on third instar larvae of *Helicoverpa armigera*, *Mythimna separata*, *S. frugiperda*, and *S. litura*. The study revealed three distinct interaction types: Type I, where Btk killed larvae too rapidly for AV infection (e.g., *H. armigera*); Type II, where mutual inhibition between Btk and AV prevented successful infection (*M. separata*/Btk + HvAV-3j and *S. litura*/Btk + HvAV-3h); and Type III, where Btk facilitated AV colonization, leading to systemic infection (*M. separata*/Btk + HvAV-3h and *S. litura*/Btk + HvAV-3h). The Type III treatments significantly reduced larval feeding and emergence rates, suggesting potential for controlling both current and future generations of these pests.

In contrast, baculoviruses, particularly nucleopolyhedroviruses (NPVs) and granuloviruses (GVs), have long been established as effective microbial insecticides. Their high oral infectivity, environmental stability, and proven success in large-scale field applications have earned them broad commercial use and regulatory approval. Like AVs, baculoviruses exhibit high host specificity and safety for non-target organisms, but their superior transmission and relatively rapid kill dynamics make them the benchmark for viral biocontrol agents in integrated pest management (IPM) programs [383].

## 11. Future Studies

**Chart 1:**
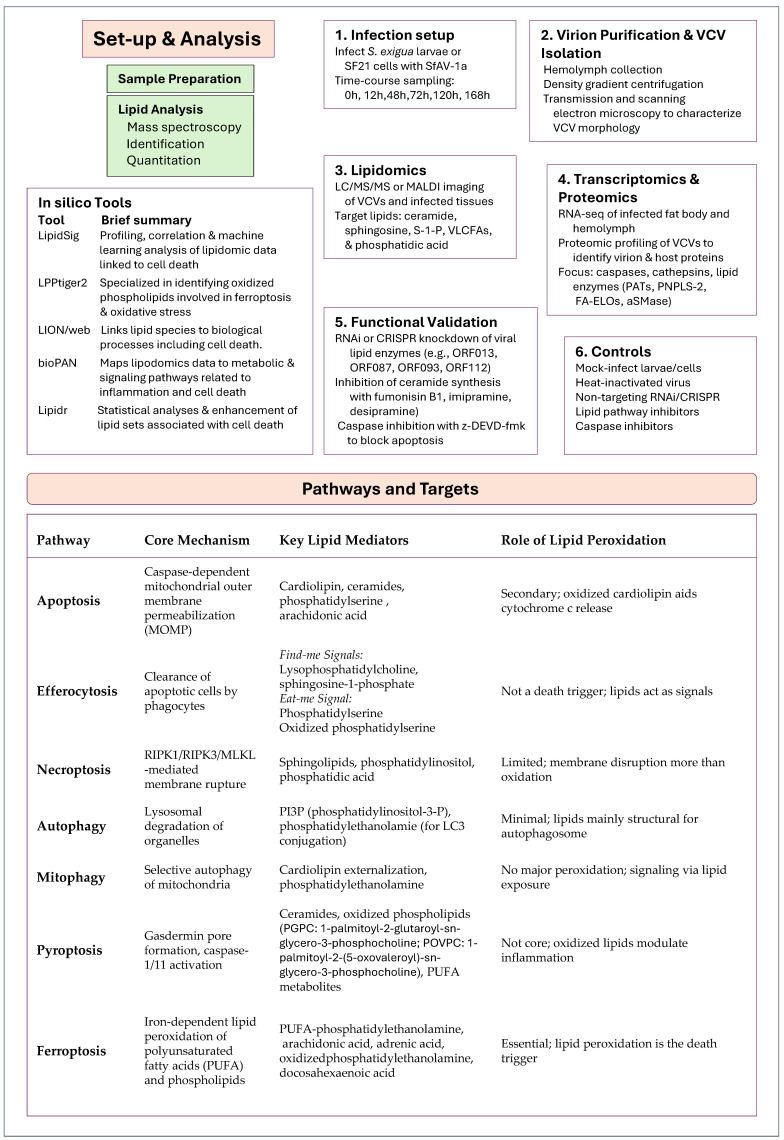
Proposed Lipidomics Workflow to Identify Candidate Pathways Involved in Ascovirus (AV) Cytopathology and Virion Containing Vesicle (VCV) Biogenesis.

To further elucidate the molecular mechanisms underlying AV cytopathology, future studies should focus on lipidomics, a powerful approach for profiling lipid species and understanding their roles in cell death reprogramming and VCV biogenesis (Chart 1). As AV cellular reprogramming is extensive (Figure 1), it is apparent that these viruses exploit host lipid biosynthetic pathways, particularly the ceramide–S-I-P rheostat (Figure 3), to modulate pyroptosis and apoptosis and promote vesicle formation [12,13]. Indeed, other cell death pathways could be involved (Figure 4). To elucidate these pathways, comprehensive lipidomic profiling of infected tissues, and purified VCVs and virions using techniques such as liquid chromatography–mass spectrometry (LC-MS/MS), tandem mass spectrometry (MS/MS), and shotgun lipidomics will help identify key lipid species, including those associated with the rheostat, cell death and survival, membrane remodeling, VCV stability, and immune evasion (Chart 1) [280,384,385,386,387,388,389,390,391,392,393]. Spatial lipidomics using MALDI imaging or Raman spectroscopy could further reveal lipid distribution within infected tissues, cells and vesicles [394].

Experimental designs should include time-resolved sampling of hemolymph and fat body tissues post-infection, isolation of VCVs via density gradient centrifugation, and integration of lipidomic data with transcriptomic and proteomic profiles. Functional validation using RNA interference or CRISPR/Cas9 [365,366] to knock down lipid-modifying enzymes (e.g., phosphate acyltransferases, patatin-like phospholipases, fatty acid elongases) will clarify their roles in VCV formation and cell death modulation. Additionally, inhibition of sphingolipid synthesis or ceramide metabolism could be used to test the dependency of AV replication on specific lipid pathways.

Challenges include the complexity of lipid extraction from insect tissues, potential contamination of VCV preparations, and the need for high-resolution lipid identification in small sample volumes. Moreover, distinguishing host-derived lipids from virally reprogrammed ones requires careful experimental controls and bioinformatic analysis. To overcome these, standardized protocols for insect lipidomics, use of internal standards, and advanced data normalization techniques should be employed [395,396]. Collaborative efforts integrating lipidomics with systems biology and imaging will be essential to build a comprehensive model of AV-induced membrane remodeling and vesicle biogenesis.

In summary, lipidomics combined with targeted functional assays offers a robust framework for decoding the biochemical landscape of AV cytopathology, as modeled in Figure 4 and Chart 1. The identification of key cellular and AV-encoded lipid-metabolic enzymes and their predicted roles in VCV formation (Table 5) suggests that AVs actively remodel host membranes to transform apoptotic bodies into metabolically active acellular compartments. In addition, Table 6 outlines a series of falsifiable predictions and experimental approaches, such as enzyme inhibition, gene knockdown, and lipid tracer assays, that directly test the contribution of both host-derived and AV-encoded lipid regulators to VCV biogenesis. These experimentally tractable hypotheses not only validate the functional relevance of the AV lipid toolkit but also provide a roadmap for dissecting virus–host interactions and developing novel biocontrol strategies.

This table summarizes the key lipid-metabolic enzymes implicated in virion-containing vesicle (VCV) biogenesis, mapped across the genomes of SfAV-1a, TnAV-6a, HvAV-3h, For each enzyme, the corresponding viral ORF, predicted catalytic domain (or protein family), and putative functional role in membrane remodeling or VCV formation are presented. This comparative resource enables gene-level mapping of the ascovirus lipid toolkit, guiding hypothesis-driven experimental validation and facilitating cross-species analyses of virus-induced cell death and vesicle biogenesis.

This table presents the key mechanistic predictions generated by the integrated model of ascovirus-driven virion-containing vesicle (VCV) formation. For each hypothesis, a recommended experimental assay is provided, facilitating targeted validation using genetic, and biochemical approaches. The roadmap supports rigorous inquiry of lipid metabolism, cell death modulation, and vesicle biology in insect virus systems, operationalizing the theoretical framework for future studies.

## 12. Conclusions

AVs offer a novel model for studying virus–host interactions and ecology due to their unique structural biology and evolutionary adaptations. Unlike conventional viruses, AVs repurpose apoptotic bodies into membrane-bound vesicles that circulate in the hemolymph, enabling virion maturation outside living cells. These vesicles maintain physiological activity by preserving mitochondrial function and deploying viral proteins, such as caspases, cathepsins, and lipid-modifying enzymes that coordinate VCV formation. Transmission of AV occurs via parasitoid wasps among lepidopteran larvae, forming a complex tripartite ecological system. Although AVs act more slowly than baculoviruses and Bt and rely on parasitoid vectors, their biocontrol potential may be enhanced through combined formulations with these well-established biocontrol agents, and through genetic optimization, such as leveraging immune-suppressive genes like ORF85, an aegerolysin-like protein in HvAV-3h. Further research into AV host specificity, tissue tropism, and vesicle biology could inform innovative pest management strategies and deepen our understanding of virus–host–parasitoid coevolution, particularly through conserved genes like the Parasitoid Killing Factor (PKF), shared across AVs, baculoviruses, and entomopoxviruses.

## Figures and Tables

**Table 1:** General properties of complete genomes of ascovirus and toursvirus isolates.

Genus	Accession	Genome	G+C	Coding
	Number	Length	(%)	Sequences *
***Ascovirus***				
Spodoptera frugiperda ascovirus 1a (SfAV-1a)	NC_008361.1	156,922	49.26	123
Trichoplusia ni ascovirus 2c (TnAV-2c)	NC_008518.1	174,059	35.24	165
Trichoplusia ni ascovirus 6b (TnAV-6b)	KY434117.1	185,664	35.43	178
Heliothis virescens ascovirus 3e (HvAV-3e)	NC_009233.1	186,262	45.88	178
Heliothis virescens ascovirus 3f (HvAV-3f)	NC_044938.1	198,157	46.0	190
Heliothis virescens ascovirus 3g (HvAV-3g)	JX491653.1	199,721	45.85	194
Heliothis virescens ascovirus 3h (HvAV-3h)	KU170628.1	190,519	45.50	185
Heliothis virescens ascovirus 3i (HvAV-3i)	MF781070.1	185,650	45.42	181
Heliothis virescens ascovirus 3j (HvAV-3j)	LC332918.1	191,718	45.62	189
***Toursvirus***				
Diadromus pulchellus toursvirus (DpTV-1a)	NC_011335.1	119,343	48.16	119
Dasineura jujubifolia tourvirus (DpTV-2a)	MK867691.1	142,600	45.97	141

* Include known and putative open reading frames typically coding for >/= 50 amino acids.

**Table 2:** Proteins with conserved domains shared by SfAV-1a, TnAV-6a and HvAV-3i *.

Protein Name	Function Summary	References
Major capsid protein (MCP)	Forms the viral capsid; facilitates host cell attachment, virion entry, assembly, and immune evasion; highly abundant in SfAV-1a, HvAV-3a, and TnAV-6a.	[75,76,77]
P64 family of DNA-binding proteins	Homologous to neurofilament H1-like proteins; condenses AV gDNA for encapsidation; contains virus-specific motifs and basic tandem repeats; cationic protein with predicted isoelectric point (pI) = 12.1; abundant and structurally unique.	[77,78,79,80,81,82,83,84,85,86]
Serine/threonine kinase (S/T-K)	Phosphorylates proteins to regulate viral replication, immune evasion, and virion assembly; may phosphorylate P64 to disrupt viral DNA complexes.	[45,77,78,82,83,84,85,86,87]
CTD phosphatase transcription factor	Dephosphorylates RNA polymerase II; manipulates host transcription to enhance viral gene expression and replication.	[88,89]
Inhibitor of apoptosis (IAP)-like protein	Suppresses host cell death; inhibits caspases, modulates ubiquitination, and disrupts immune signaling to prolong cell survival.	[90,91]
SNF2 DEAD-like helicase	Unwinds DNA/RNA using ATP hydrolysis; essential for viral genome replication, repair, and recombination.	[92,93]
Myristylated membrane protein-like protein	Targets viral proteins to membranes via lipid modification; involved in viral entry, assembly, budding, and egress.	[94,95,96]
Sulfhydryl oxidase Erv1-like protein	Catalyzes disulfide bond formation; ensures proper folding and stability of viral proteins and virion integrity.	[97,98,99]
S1/P1 nuclease	Degrades single-stranded DNA/RNA; facilitates genome processing, nucleic acid maturation, and host defense evasion.	[100,101,102]
Dynein-like β chain	Motor protein subunit; drives microtubule sliding, mitotic spindle positioning, chromosome segregation, and cargo transport.	[103,104,105,106,107,108]

* Additional data provided in “Supplementary Materials”.

**Table 3:** SfAV-1a and other ascovirus and toursvirus protein homologs and function.

SfAV-1a Gene	Virus	Protein Description
DNA metabolism		
ORF001	H, T, D	DNA polymerase
ORF037	T, D	GIY_YIG-like endonuclease
ORF066	H, T	FLAP-like endonuclease
ORF059	H, T, D	DNA repair exonuclease
ORF103	H, T, D	ATPase involved in DNA repair
ORF110	H, T, D	ATPase
ORF099	H, T, D	Primase
ORF095	H, T	Helicase 2
ORF086	H, D	Uvr/REP helicase
RNA metabolism		
ORF008	H, T, D	DNA-directed RNA polymerase largest subunit, N-terminal domain
ORF052	H, T, D	DNA-directed RNA polymerase subunit B
ORF067	H, T, D	DNA-directed RNA polymerase largest subunit, C-terminal domain
ORF022	H, T, D	RNase III
ORF113	H, T, D	VLTF2-like late transcription factor/Zn finger DNA binding protein
Nucleotide metabolism		
ORF040	H, T, D	Thymidine kinase
ORF090	H, T, D	Putative lipopolysaccharide-modifying enzyme/tyrosine protein kinase
ORF104	H, T, D	Serine/threonine kinase-like protein
Inhibitor of Apoptosis-Like Proteins		
ORF016	H, T, D	RING Finger, E3 ubiquitin ligase
ORF025	H, T, D	RING Finger, E3 ubiquitin ligase
ORF074	H, T	RING Finger, E3 ubiquitin ligase
Apoptosis		
ORF073	H, T	Caspase or Caspase-like/Interleukin-1-β–converting enzyme
ORF114	H, T, D	Cathepsin B
Lipid Metabolism		
ORF013	H, D	Esterase/Lipase
ORF087	H, T, D	Fatty acid elongase
ORF093	H, T, D	Patatin-like phospholipase A2 (PLA2)
ORF112	H, T, D	Phosphate acetytransferase

Ascovirus (AV) isolates: H, Heliothis virescens AV; T, Trichoplusia ni AV; D, Diadromus pulchellus AV Zaghloul et al. [18].

**Table 4:** Ascovirus-coded enzymes and their putative roles in lipid metabolism, regulated cell death pathways, and biogenesis of virion containing vesicles (VCV) *.

Enzyme	Selected Ascoviruses (ORF/Protein ID)	Cellular Function	Role in Cell Death and VCV Biogenesis
Esterase/Lipase	SfAV-1a: 013/YP_762368.1 TnAV-6b: 139/AUS94238.1 HvAV-3i: 018/AXN77201.1	Hydrolyzes ester bonds in lipids, releasing fatty acids	May generate free fatty acids that undergo lipid peroxidation, a key trigger of ferroptosis. Also supports viral replication, cell cleavage, and VCV formation, potentially enhance infectivity through membrane remodeling.
Fatty Acid Elongase (FA-ELO)	SfAV-1a: 087/YP_762442.1 TnAV-6b: 053/AUS94152.1 HvAV-3i: 141/AXN77324.1	Extends fatty acid chains to produce very-long-chain fatty acids (VLCFAs)	VLCFAs can disrupt membrane integrity via interdigitation, promoting necroptosis. May alter host membrane composition, aiding immune evasion and viral replication. VLCFAs may also contribute to VCV biogenesis and stability.
Patatin-like Phospholipase A2 (PNPLA)	SfAV-1a: 093/YP_762448.1 TnAV-6b: 073/AUS94172.1 HvAV-3i: 128/AXN77311.1	Hydrolyzes phospholipids, releasing lysophospholipids and fatty acids	Facilitates organelle interactions and intercellular spread, possibly contributing to host cell remodeling, including membrane curvature and VCV biogenesis. May induce ER stress and disrupt mitochondrial integrity. Reactive oxygen and nitrogen species released from mitochondria can promote lysosomal membrane permealization leading to the release of acid hydrolases including cathepsin and acid sphingomyelinase (aSMase) which can activate the intrinsic apoptotic response.
PlcS, Phosphate Acyltransferase (PAT)	SfAV-1a: 112/YP_762467.1 TnAV-6b: 105/AUS94204.1 HvAV-3i: 107/AXN77290.1	Transfers acyl groups to phospholipids, modifying membrane composition	Regulates incorporation of polyunsaturated fatty acids (PUFAs) into membranes which can either promote or protect against ferroptosis, depending on lipid context. May be involved in modulating membrane fluidity and viral envelope and VCV biogenesis.
Caspase/Caspase-like protein	SfAV-1a: 073/YP_762428.1 TnAV-6b: 79/AUS94178.1 HvAV-3i: 167/AXN77350.1	Proteolytic enzymes that cleave substrates during apoptosis	Central regulators of apoptosis. May assist in host cell remodeling and viral protein processing, and precursor events leading to VCV biogenesis.
Cathepsin B	SfAV-1a: 114/YP_762469.1 TnAV-6b: 109/AUS94208.1 HvAV-3i: 103/AXN77286.1	Lysosomal protease involved in protein degradation	May trigger controlled cell death under stress. Potentially activates viral caspase and contributes to cellular degradation.

* Refer to Figure 4 and text for further information on the predicted functions of these enzymes in ascovirus biology.

**Table 5:** Lipid-Metabolic Toolkit of Major Ascoviruses (AVs).

Enzyme/Class	Catalytic Domain/Family	SfAV-1a ORF	TnAV-6a ORF	HvAV-3h ORF	Predicted Role in VCV Formation
Acid sphingomyelinase (aSMase)	Sphingomyelinase, (SMPD)	ORF085	ORF38	ORF29	Ceramide microdomain formation, membrane curvature
Sphingosine kinase (SphK)	Sphingosine kinase, (SphK)	ORF091	ORF50	ORF47	S-1-P production, regulation of apoptosis/survival
Fatty acid elongase (FA-ELO)	FA elongase (GNS1/SUR4)	ORF033	ORF12	ORF04	VLCFA synthesis, ceramide and lipid precursors
Patatin-like phospholipase (PNPLA-2)	PNPLA (Patatin)	ORF017	ORF11	ORF16	Phospholipid hydrolysis, membrane remodeling
Esterase/Lipase	Lipase/esterase	ORF019	ORF15	ORF19	Lipid turnover, VCV cleavage, virion assembly
Ceramide synthase	Ceramide synthase	ORF079	ORF41	ORF05	Ceramide production, membrane biogenesis
Chitinase/Cathepsin Suppressor	Cathepsin inhibitor, Chitinase suppressor	ORF32	ORF26	ORF31	Limits host tissue liquefaction to support VCV persistence
Phosphate acyltransferase (PAT)	PlsC family (GPAT/AGPAT)	ORF029	ORF20	ORF21	Glycerophospholipid biosynthesis

**Table 6:** Falsifiable Predictions and Experimental Assays for Ascovirus Lipid-Centric VCV Biogenesis.

Hypothesis	Falsifiable Test	Experimental Approach
VCV biogenesis is dependent on host-derived aSMase activity and ceramide microdomain formation	Inhibiting aSMase function will reduce VCV formation and alter vesicle membrane composition	Treat infected insect cells/larvae with imipramine or desipramine (aSMase inhibitors); quantify VCVs and analyze membrane lipids via LC-MS/MS
Sphingosine-1-phosphate (S-1-P) balance modulates VCV structure/function via SphK activity	SphK inhibitor SKI-II disrupts normal ceramide/S-1-P ratio, resulting in abnormal VCV morphology or reduced virus yield	Apply SKI-II to infected cultures; quantify S-1-P and ceramides by lipidomics; assess VCV integrity and infectious output
Caspase inhibition at late stages impairs VCV cleavage and virion dissemination	Pharmacological or RNAi-mediated inhibition of executioner caspases at late infection reduces VCV partitioning and virion release	Apply caspase inhibitors or RNAi at different time points; monitor VCV biogenesis and release by microscopy and titration
Functional mitophagy supports proper VCV formation	Interfering with mitophagy pathways alters mitochondrial positioning or reduces VCV biogenesis	Use mitophagy probes (mito-Keima, mCherry-GFP-Atg8) or Parkin/FUNDC1 immunostaining; assess changes in mitochondrial localization and VCV cleavage
VCVs evade efferocytosis by lacking surface “eat-me” signals or overwhelming clearance machinery	VCVs exposed to annexin V show altered hemocyte uptake kinetics; blocking PS exposure impairs VCV clearance	Flow cytometry for PS exposure (Annexin V) on VCVs; track VCV uptake by hemocytes in vitro with/without masking treatments
Lipidomic shifts in infected tissues predict changes in VCV composition and infectivity	Time-course analysis reveals ceramide and S-1-P enrichment in VCVs compared to uninfected controls	Extract and analyze lipids from infected tissues and isolated VCVs (LC-MS/MS) at multiple infection time points
Cathepsin/chitinase suppression by viral ORFs modulates host tissue dissolution and VCV yield	Overexpression or knockout of HvAV-3h ORF31 changes substrate breakdown and the abundance of VCVs	Use gene perturbation assays; quantify protease activity and VCVs

## Data Availability

Not applicable.

## Supplementary Materials

The following supporting information can be downloaded at: https://www.mdpi.com/article/10.3390/pathogens14111094/s1, Table S1: SfAV-1a virion protein homologs.
