# Supplementary material for: Pathogens That Rewrite the Rules: Ascoviruses, Elegant Manipulators of Cell Death Pathways and Architects of the Extracellular Viral Paradigm
Source: Pathogens. 2025 Oct 27;14(11):1094. doi: 10.3390/pathogens14111094 (PMC12655742; doi:10.3390/pathogens14111094)
Supplement: Supplementary file 1 [file pathogens-14-01094-s001.zip › pathogens-3863183-supplementary.pdf]

Supplementary Materials: TABLE S1

SfAV-1a ORF (kDa)	TnAV-6a ORF	HvAV-3i ORF	Putative and Known Function
2 (13.7)	n	3	hypothetical protein; function unknown
3 (25.4)	n	4	hypothetical protein; function unknown
9 (110.8)	161	14	SNF2 DEAD-like helicase
15 (64.2)	6	20	Inhibitor of apoptosis (IAP)-like protein
27 (77.2)	11	28	hypothetical protein; function unknown
33 (27.5)	135	43	hypothetical protein; function unknown
35 (25.6)	157	n	hypothetical protein; function unknown
36 (20.1)	156	49	hypothetical protein; function unknown
38 (22.2)	155	51	hypothetical protein; function unknown
41 (50.1)	152	54	Major capsid protein
43 (26.0)	148	56	hypothetical protein; function unknown
47 (104.9)	n	n	DNA puffC4B-like protein
48 (64.6)	141	59	Ascovirus P64 family of DNA binding proteins; Neurofilament triplet H1-like
54 (30.3)	129	63	Myristylated membrane protein-like protein
60 (8.6)	n	70	hypothetical protein; function unknown
61 (25.3)	118	72	Sulfhydryl 1 oxidase Erv 1-like protein
64 (90.0)	114	76	Serine/threonine protein kinase
75 (29.6)	135	86	S1/P1 nuclease
84 (119.3)	43	147	Dynein-like β chain
91 (14.4)	59	130	HMG_Box/Yabby-like transcription factor
109 (22.4)	93	111	CTD phosphatase transcription factor

kDa, kilodaltons; n, SfAV-1a homolog absent; ORF, open reading frame;
ORFs shown in bold type code for proteins with known functions or conserved domains.

(i) *Major capsid protein (MCP)***.** Apart from its role in forming a protective shell (capsid) around the viral genomes, the MCP also aids in the attachment and entry of the virion into host cells and the proper assembly of new virions. Capsid proteins also play roles in evading the host immune response [76,77]. AV MCP proteins together with the P64 family of proteins (below) are the two most abundant virion proteins, each occurring at molar ratios of >10 compared to all other virion proteins of SfAV-1a, HvAV-3a, and TnAV-6a [78].

(ii) *AV P64 family of DNA-binding proteins (neurofilament triplet H1-like proteins).* The P64 family of proteins [78,79] show a significant level of homology with neurofilament triplet H1-like proteins, which are the most abundant structural components of the neuronal cytoskeleton [80,81]. SfAV-1a P64, a highly cationic protein (*pI* = 12.1), is one of the few AV proteins that has been characterized structurally and functionally [78,79]. This protein has a novel bipartite architecture not known to occur in other proteins. P64 contains four copies of a virus-specific 2-cysteine adaptor (vs2C-ad) motif (residues 1–219; pfam08793; cl07414) [82] with an intervening stretch of basic amino acids (95-RGTSPSRRSRSRSMSPRRRASPARRR-112) between two vs2C-ad in the N-

terminal domain (residues 1–263), and 14 tandem repeats of an arginine/serine-rich motif [SPSQRRSTS(V/K)(A/S)RR] in the C-terminal domain (residues 279–455).

P64 progressively localizes from the virogenic stroma into the virion core, and physical evidence (electrophoretic mobility shift assay and transmission electron microscopy) demonstrates the protein, and its domains and motifs when assayed independently, condense SfAV-1a gDNA. Based on these findings, it was proposed that P64 and its homologs comprise a novel family of atypical large basic proteins that condense AV gDNA for encapsidation [78]. The functional role of the P64 family of proteins is another unique feature of AVs, as other viruses typically use divalent cations, polyvalent protamine cations such as spermine and spermidine, or small basic proteins rich in lysine and arginine, including protamine-like and histone-like proteins, generally <15 kDa, to condense genomic DNA for encapsidation [83–87].

(iii) Serine/threonine kinase (S/T-K). S/T-Ks are essential in viral biology as they phosphorylate serine or threonine residues in proteins, altering their function, location, and intermolecular interactions [88]. These enzymes regulate viral replication by modifying both viral and host proteins to favor propagation of the pathogen. They also help the virus evade the host's immune response by interfering with immune signaling pathways. Additionally, S/T-Ks control the assembly of viral components and the release of new virions from infected cells.

The potential of S/T-K to directly interact with virion structural proteins is intriguing. In a previous study [46], we demonstrated that the capsid protein was the only phosphorylated protein in the SfAV-1a virion. It is unknown whether S/T-K phosphorylates the capsid. Interestingly, P64 is unphosphorylated in the virion. Yet, when P64 is produced in insect cells, it becomes heavily phosphorylated [78,79]. Since no other kinase was identified in the SfAV-1a virion, it is possible that S/T-K phosphorylates P64, leading to the uncoupling of gDNA-P64 complexes by electrostatic repulsion due to the negative charges on phosphate residues in both components.

(iv) CTD phosphatase transcription factor. CTD phosphatases dephosphorylate the C-terminal domain of RNA polymerase II, regulating transcription. Viral CTD phosphatases manipulate host transcription to favor viral gene expression, ensuring efficient replication and production of viral proteins [89,90].

(v) Inhibitor of apoptosis (IAP)-like protein. IAPs are evolutionarily conserved regulators that many viruses exploit to suppress host cell death and enhance replication. Originally identified in baculoviruses, viral IAPs mimic or stabilize host IAPs to block apoptosis, often by inhibiting caspases, modulating ubiquitination through RING domains, and interfering with immune signaling pathways like NF-κB and MAPK [91,92]. Collectively, these mechanisms underscore the intricate host-virus

interplay, where viral IAPs prolong cell survival to facilitate viral propagation.

(vi) SNF2 DEAD-like helicase. These helicases are part of the SF2 superfamily and are involved in unwinding DNA or RNA helices using energy from ATP hydrolysis. In viral replication, they play crucial roles by unwinding the viral genome, facilitating replication, and assisting in the repair and recombination of viral DNA [93,94]

(vii) Myristylated membrane protein-like protein. Myristylation is a lipid modification that targets proteins to cellular membranes. Viral myristylated proteins are involved in membrane metabolism, and viral entry, assembly, budding, and egress [95–97].

(viii) Sulfhydryl 1 oxidase Erv 1-like protein. Sulfhydryl oxidases catalyze the formation of disulfide bonds in proteins, crucial for their proper folding and stability. In viruses, these enzymes are essential for the correct assembly of viral proteins and the formation of infectious virions, maintaining the structural integrity of viral particles [98–100].

(ix) S1/P1 nuclease. This enzyme degrades single-stranded DNA and RNA [101]. In viral biology, S1/P1 nucleases are involved in processing viral genomes, removing damaged nucleic acids, facilitating the maturation of viral RNA and DNA, and in helping the virus evade host defenses by degrading host-specific nucleic acids [102,103].

(x) Dynein-like β chain. Dynein is a complex motor protein that moves along microtubules in cells, converting chemical energy from ATP into mechanical work [104]. The β chains of dynein, which are part of its heavy chain structure, play crucial roles in its function. In axonemal dyneins, which are found in cilia and flagella, the β chains contribute to the sliding of microtubules, enabling the beating motion of these structures. In mitosis, dynein β chains play a role in positioning the mitotic spindle and segregating chromosomes during cell division. Finally, in organelle transport, the β chains of cytoplasmic dyneins are involved in transporting various cellular cargos, such as organelles, vesicles, and proteins, towards the minus end of microtubules.

In viral biology, the dynein β chain is crucial for the intracellular transport of viral components within the host cell [105–109]. Viruses hijack the dynein motor complex to move towards the nucleus for replication and assembly. Dynein also aids in the disassembly of viral particles in the cytosol, facilitating their entry into the nucleus, and also in viral egress. Additionally, the dynein complex interacts with various adaptor proteins, highlighting the dynamic interplay between viruses and the host's intracellular transport machinery.

(xi) HMG_box Yabby-like transcription factor. HMG-box proteins, common in plants, are DNA-binding proteins that regulate gene expression. In viruses,

these transcription factors modulate the expression of viral genes, ensuring the timely production of proteins necessary for replication and assembly. They also interact with host cell transcription machinery to enhance viral gene expression [110–112].
